# High genetic similarity between non-typhoidal *Salmonella* isolated from paired blood and stool samples of children in the Democratic Republic of the Congo

**DOI:** 10.1371/journal.pntd.0008377

**Published:** 2020-07-02

**Authors:** Marie-France Phoba, Barbara Barbé, Benedikt Ley, Sandra Van Puyvelde, Annelies Post, Wesley Mattheus, Stijn Deborggraeve, Octavie Lunguya, Jan Jacobs

**Affiliations:** 1 Department of Microbiology, Institut National de Recherche Biomédicale, Kinshasa, Democratic Republic of the Congo; 2 Department of Microbiology, University Hospital of Kinshasa, Democratic Republic of the Congo; 3 Department of Clinical Sciences, Institute of Tropical Medicine, Antwerp, Belgium; 4 Department of Biomedical Sciences, Institute of Tropical Medicine, Antwerp, Belgium; 5 Wellcome Sanger Institute, Wellcome Genome Campus, Hinxton, Cambridge, United Kingdom; 6 Department of Human Bacterial Diseases, Sciensano, Brussels, Belgium; 7 Department of Microbiology, Immunology and Transplantation, KU Leuven, Leuven, Belgium; Mohammed Bin Rashid University of Medicine and Health Sciences, UNITED ARAB EMIRATES

## Abstract

**Background:**

Non-typhoidal *Salmonella* (NTS) serotypes Typhimurium and Enteritidis are a major cause of bloodstream infections in children in sub-Saharan Africa but their reservoir is unknown. We compared pairs of NTS blood and stool isolates (with the same NTS serotype recovered in the same patient) for genetic similarity.

**Methods:**

Between November 2013 and April 2017, hospital-admitted children (29 days to 14 years) with culture-confirmed NTS bloodstream infections were enrolled in a cross-sectional study at Kisantu Hospital, DR Congo. Stool cultures for *Salmonella* were performed on a subset of enrolled children, as well as on a control group of non-febrile hospital-admitted children. Pairs of blood and stool NTS isolates were assessed for genetic similarity by multiple-locus variable-number of tandem repeats (MLVA) and genomics analysis.

**Results:**

A total of 299 children with NTS grown from blood cultures (Typhimurium 68.6%, Enteritidis 30.4%, other NTS 1.0%) had a stool sample processed; in 105 (35.1%) of them NTS was detected (Typhimurium 70.5%, Enteritidis 25.7%, other NTS 3.8%). A total of 87/105 (82.9%) pairs of blood and stool NTS isolates were observed (representing 29.1% of the 299 children). Among 1598 controls, the proportion of NTS stool excretion was 2.1% (p < 0.0001). MLVA types among paired isolates were identical in 82/87 (94.3%) pairs (27.4% of the 299 children; 61/66 (92.4%) in Typhimurium and 21/21 (100%) in Enteritidis pairs). Genomics analysis confirmed high genetic similarity within 41/43 (95.3%) pairs, showing a median SNP difference of 1 (range 0–77) and 1 (range 0–4) for Typhimurium and Enteritidis pairs respectively. Typhimurium and Enteritidis isolates belonged to sequence types ST313 lineage II and ST11 respectively.

**Conclusion:**

Nearly 30% of children with NTS bloodstream infection showed stool excretion of an NTS isolate with high genetic similarity, adding to the evidence of humans as a potential reservoir for NTS.

## Author summary

Non-typhoidal *Salmonella* (NTS) is an important cause of bloodstream infections in sub-Saharan Africa, but little is known about its reservoir and transmission. We assessed the possibility of a human reservoir by culturing stool samples from children with NTS found in their blood. Next, we compared the obtained blood and stool NTS isolates for genetic similarity. We found that a high proportion of children with NTS in their blood also had NTS in their stool (35.1%) when compared to a control group of children without suspicion of bloodstream infection (2.1%). We observed that *Salmonella* Typhimurium and *Salmonella* Enteritidis were the main serotypes isolated from blood and stool, and that their proportions were similar within the different populations. We found that 94.3% of the paired NTS blood and stool isolates (or 27.4% of children with NTS in their blood and a stool culture done) were genetically similar to each other. This observation adds to the evidence of a potential human reservoir for NTS.

## Introduction

Non-typhoidal *Salmonella* (NTS) is a leading cause of bloodstream infections (BSI) with an estimated global burden of invasive NTS infections of 535,000 cases and a case fatality rate of 14.5% in 2017. The burden is highest in sub-Saharan Africa (sSA) and in children under five years of age [1]. *Salmonella enterica* subspecies *enterica* serotypes Typhimurium and Enteritidis are the most common causes of NTS BSI [2–5]. The pathogenicity of NTS differs between high-income countries and sSA with a higher risk of invasive disease and associated mortality in the latter [6]; this can be partly explained by the emergence of distinct and invasive clades of Typhimurium and Enteritidis serotypes across sSA [7–10]. Unlike *Salmonella* Typhi for which the role of human carriers is pivotal in the transmission [3,11], little is known about the reservoirs and transmission of NTS in sSA, but environmental, zoonotic and human sources have been hypothesized [3,12,13]. The role of NTS excretion in the stool has not yet been elucidated and it is unknown whether the NTS strains in the blood of BSI patients are also excreted in stool [3,13].

The objectives of this study were to determine the proportion of NTS stool excretion among hospital-admitted children with NTS BSI and non-febrile hospitalized children as a control group, and to assess the genetic similarity of paired blood and stool NTS isolates *(i*.*e*. identical NTS serotypes obtained from the same patient in the course of the hospital admission).

## Methods

### Study site and study period

The study took place from November 2013 to April 2017 at the hospital of Saint-Luc in Kisantu (HSLK), Kongo-Central province, Democratic Republic of the Congo (DR Congo). HSLK is the major sampling site of the microbiological surveillance network organized by the Institut National de Recherche Biomédicale (INRB), Kinshasa, DR Congo, and the Institute of Tropical Medicine, Antwerp, Belgium [4,14]. Over a 10-year period (2007–2017), NTS ranked first as a cause of BSI in children accounting for 63.8% of culture-confirmed BSI [14,15].

### Study design

A cross-sectional study in which hospital-admitted patients aged 29 days to 14 years old with culture-confirmed NTS BSI were enrolled (further referred to as “NTS BSI group”). Enrollment depended on the availability of regular clinical staff during office hours. As soon as possible after enrollment, a stool sample was collected for culture. Stool cultures were also sampled in a control group selected by convenience and consisting of non-febrile hospital-admitted children, as they were presumed to be most closely related to the NTS BSI group in terms of demographics and residency or referring health center. A single stool culture was sampled per patient–repeat cultures were removed from analysis and only the first NTS isolate was considered.

### Blood and stool cultures

Blood cultures were collected and processed as previously described [4,15]. About 1 g of stool was suspended in 10 ml selenite broth (Oxoid, Basingstoke, UK and BD Difco^TM^, Becton, Dickinson and Company, Franklin Lakes, NJ, U.S.), and incubated at 36°C for 18–24 hours. Next, 10 μl of selenite broth was inoculated on two plates of Salmonella-Shigella (SS) agar (Oxoid) which were incubated at 36°C for 18–24 hours. In case of absence of growth on the SS plate, the plate was re-incubated for another 24 hours. Two colonies suspected of *Salmonella* per SS plate were inoculated on a Kligler Iron Agar (KIA) tube (Oxoid) and incubated at 36°C for 18–24 hours. KIA tubes suggestive of *Salmonella* species were further identified by their biochemical characteristics [2,16]. *Salmonella* spp. isolates from blood and stool were stored in tubes with Tryptone Soy Agar (Oxoid) and shipped to INRB for serotyping and ITM for confirmation and molecular testing. *Salmonella* spp. isolates were serotyped using commercial antisera (Sifin, Berlin, Germany; and Vision^TM^, Pro-Lab Diagnostics, Richmond Hill, Canada) according to the Kaufman-White scheme [17].

### Multiple-locus variable-number of tandem repeats analysis (MLVA)

MLVA typing was performed at Sciensano (Brussels, Belgium) on *Salmonella* Typhimurium and *Salmonella* Enteritidis isolates from blood and stool. MLVA typing was performed using a 5-loci MLVA scheme for both serotypes (STTR9-STTR5-STTR6-STTR10-STTR3 for *Salmonella* Typhimurium and SENTR7-SENTR5-SENTR6-SENTR4-SE-3 for *Salmonella* Enteritidis) [18,19]. MLVA types were reported as a string of five numbers representing the number of repeats at the corresponding locus or “NA” in case a PCR product was not obtained for that locus.

### Whole genome sequencing (WGS) and maximum likelihood phylogenetic tree analysis

A subset of NTS pairs recovered during the first months of the study (see further) were subjected to whole-genome sequencing (listed in S1 Table). Total DNA was extracted using the Gentra Puregene extraction kit (Qiagen, Hilden, Germany), a sequencing library was prepared using the TruSeq library prep kit (Illumina, San Diego, CA, USA) and sequencing was done on the Illumina 1500 HiSeq platform at the University of Antwerp sequencing facility (Belgium). *Salmonella* Typhimurium and *Salmonella* Enteritidis sequences were respectively mapped to the *Salmonella* Typhimurium D23580 (Accession Number = NC_016854) and *Salmonella* Enteritidis P125109 (Accession Number = AM933172) reference sequences, using SMALT v0.7.4. Variation detection was performed using samtools mpileup v0.1.19 (-d 1000 -DSugBf) and bcftools v0.1.19 [20]. Per serovar, a pseudo-genome was constructed by substituting the base call at each site in the BCF file into the reference genome. Uncertain sites were substituted with an N. Insertions with respect to the reference genome were ignored and deletions with respect to the reference genome were filled with Ns in the pseudo-genome. Plasmids and recombinant regions (prophages and CRISPR sequences) were detected using Phaster and Gubbins v1.4.10 and were removed from the core genome alignment [21–23]. Single nucleotide polymorphism (SNP) sites were extracted using snp-sites and used to construct a maximum likelihood phylogeny with RAxML v8.2.8 with substitution model GTRCAT [24,25]. Support for nodes on the trees was assessed using 1000 bootstrap replicates. Trees were rooted on *Salmonella* Typhi 10040_15 (Accession Number = ERS1574281) [26]. The number of SNPs on the branches was calculated using Sankoff Parsimony [27–29]. Phylogenetic trees were visualized using iTOL [30].

### Definitions

Paired NTS isolates (NTS pairs) were defined as corresponding blood and stool NTS isolates with identical serotype recovered from the same patient. Co-presence was defined as corresponding blood and stool NTS isolates with different serotypes recovered from the same patient. Identical MLVA types for the Typhimurium serotype were defined as isolates with variation in none or one of the rapidly changing loci (STTR5, STTR6 and STTR10) and no variation in the stable loci (STTR3 or STTR9) [31]; identical MLVA types for the Enteritidis serotype were defined as isolates with variation in none or one of the five loci [32]. For WGS, genetic similarity was based on the relatedness of the paired isolates in the phylogenetic tree.

### Data analysis

Data were encoded into an Excel database (Microsoft, Redmond, Washington). Data were characterized by proportions, percentages, ratios, medians, 25–75% interquartile ranges (IQR) and ranges. Differences between proportions were tested for significance using the χ2 test or the McNemar’s test in case of correlated proportions; medians were compared using the Mann-Whitney U test. A p-value of < 0.05 was considered significant.

### Ethical issues

Ethical approval was granted by the Institutional Review Board of the Institute of Tropical Medicine in Antwerp (Belgium), the Ethics Committees of the University of Antwerp (Belgium) and the School of Public Health of the University of Kinshasa (DR Congo). Oral informed consent was requested from the guardian of the participants; additional oral assent was requested from participants of 12 years and older. The reasons for oral rather than written consent included the absence of potential risk and individual benefit of stool collection and analysis (non-invasive, no relevance for patient care) as well as the potential impact on the already stressful situation of the participant and his/her caretakers and the need for timely antibiotic treatment. The use of oral consent was approved by the above mentioned ethical committees. A logbook at the pediatric ward was used to document the oral consent.

## Results

Among 1052 children with NTS grown from blood cultures, 299 (28.4%) had a stool sample processed (Table 1). Their median age (available for 298/299 (99.7%) children) was 1.5 (IQR 0.9–2.6) years; 78.2% (233/298) were < 36 months and 92.3% (275/298) were < 5 years old. The control group consisted of 1598 children; their median age (available for 100% of children) was 1.8 (IQR 0.9–3.8) years. The median age between both groups did not differ significantly (p = 0.02) and the male-to-female ratio was identical (1:0.9). Overall, the most common diagnoses (n = 4349) of children admitted for non-febrile diseases during the study period were sickle cell anemia (31.6%), malnutrition (23.4%) and amoebiasis (6.4%). Patient inclusion was consistent over the study period, varying between 19.8% (2014) and 36.8% (2013) respectively (S1 Table).

**Table 1:** Proportions and serotype distributions of non-typhoidal *Salmonella* blood and stool isolates among children with culture-confirmed bloodstream infection versus a control group of non-febrile hospital-admitted children.

	NTS blood isolates			NTS stool isolates		
**NTS serotype**	All children with NTS BSI (n = 1052)	Enrolled children with NTS BSI and stool culture done (NTS BSI group) (n = 299)	p-value	Enrolled children with NTS BSI and NTS stool excretion (n = 105)	Control group (n = 1598) with NTS stool excretion (n = 34)	p-value
Typhimurium	645 (61.3%)	205 (68.6%)	0.001	74 (70.5%)	25 (73.5%)	0.98
Enteritidis	402 (38.2%)	91 (30.4%)		27 (25.7%)	9 (26.5%)	
Other NTS	5* (0.5%)	3^$^ (1.0%)		4^±^ (3.8%)	0 (0.0%)	
**NTS stool excretion**	NA	NA		105/299 (35.1%) (95% CI: 29.8–40.9%)	34/1598 (2.1%) (95% CI: 1.5–3.0%)	<0.0001

*1 *Salmonella* Aberdeen, 1 *Salmonella* Abony, 1 *Salmonella* Chandans, 1 *Salmonella* Stanleyville, 1 *Salmonella* Urbana

^$^1 *Salmonella* Aberdeen, 1 *Salmonella* Stanleyville and 1 *Salmonella* non-typable isolate

^±^2 *Salmonella* Virchow and 2 *Salmonella* non-typable isolates. Abbreviations: BSI = bloodstream infection, CI = confidence interval, NA = not applicable, NTS = non-typhoidal *Salmonella*.

Table 1 lists the proportions and serotype distributions of the NTS stool isolates. NTS stool excretion in the NTS BSI group was 35.1% versus 2.1% in the control group (p < 0.0001). NTS serotypes included mainly Typhimurium and Enteritidis, with the former being the most frequent. Serotype distributions were similar among blood cultures and stool cultures from both NTS BSI and the control group; however, a small but significant difference (p = 0.001) was found between the total NTS BSI group and the children with NTS BSI and a stool culture done. The proportions of stool cultures grown did not differ significantly between the dry and the rainy season (36.8% (21/57) versus 34.7% (84/242) respectively, p = 0.84) (S1 Table).

Table 2 lists the serotype distributions of the NTS blood and stool isolates from 299 children with a NTS BSI, of whom 105 (35.1%) had NTS isolated from both blood and stool. In 87 of these children (87/105, 82.9%; 87/299, 29.1%), pairs of blood and stool NTS were recovered; representing 66/74 (89.2%) and 21/27 (77.8%) of the Typhimurium and Enteritidis stool culture isolates respectively. In the remaining 18/105 (17.1%) children, serotypes of NTS blood and stool isolates were different ("co-presence"). The median delay between blood and stool sampling within the NTS pairs and NTS co-presence was 2 days and 1.5 day respectively (IQR of 1–3 days). For 9.2% (8/87) of the NTS pairs, stool sampling was done before or on the same day as the blood sampling (1 and 7 pairs respectively). Of note, two NTS pairs had a delay between blood and stool sampling of more than 14 days (16 days and 43 days); the corresponding patients had extended hospital stays because of suspicion of tuberculosis and treatment for malnutrition. (S2 Table)

**Table 2:** Serotype distribution of non-typhoidal *Salmonella* blood and stool isolates from 299 children with non-typhoidal *Salmonella* bloodstream infection.

	Stool cultures				
Blood cultures	Typhimurium	Enteritidis	Other NTS	No growth of *Salmonella*	Total
Typhimurium	66*	6^‡^	1^‡^ ^±^	132	205
Enteritidis	8^‡^	21*	3^‡^ ^†^	59	91
Other NTS	0	0	0	3^$^	3
Total	74	27	4	194	299

The grey shaded cells represent all non-typhoidal *Salmonella* isolates cultured from stool in children with NTS BSI (= 105/299, 35.1%).

*Paired NTS isolates

^‡^Co-presence of non-identical NTS serotypes

^±^1 *Salmonella* non-typable isolate

^†^2 *Salmonella* Virchow and 1 *Salmonella* non-typable isolate

^$^1 *Salmonella* Aberdeen, 1 *Salmonella* Stanleyville and 1 *Salmonella* non-typable isolate. Abbreviations: BSI = bloodstream infection, NTS = non-typhoidal *Salmonella*.

MLVA types were identical in 61/66 (92.4%) Typhimurium and 21/21 (100%) Enteritidis pairs; for both serotypes combined, this proportion was 94.3% (82/87) (Tables 3 and 4), representing 27.4% of 299 children with NTS BSI in whom stool cultures were done. The two Typhimurium pairs with a delay between blood and stool sampling of 16 days and 43 days had identical MLVA types (S2 Table). MLVA types of the stool NTS recovered from the control group were similar to those of the NTS pairs (S3 Table).

**Table 3:** Overview of MLVA types of *Salmonella* Typhimurium paired isolates recovered from stool versus blood.

	Typhimurium MLVA types from blood																														
Typhimurium MLVA types from stool	2-4-12-7-0210	2-5-10-7-0210	2-5-11-7-0210	2-5-13-8-0210	2-5-14-8-0210	2-5-15-8-0210	2-5-16-8-0210	2-6-7-9-0210	2-6-9-9-0210	2-6-18-9-0210	2-6-NA-9-0210	2-7-14-6-0210	2-7-15-6-0210	2-7-9-9-0210	2-8-10-8-0210	2-8-11-8-0210	2-8-11-NA-0210	2-8-12-8-0210	2-8-12-9-0210	2-8-14-8-0210	2-8-14-9-0210	2-9-11-7-0210	2-9-11-8-0210	2-9-12-7-0210	2-NA-9-7-0210	3-7-13-8-0210	3-7-14-8-0210	3-NA-10-7-0210	3-NA-12-7-0210	3-NA-7-7-0210	Total
2-4-12-7-0210	0					1*																									1
2-5-10-7-0210		3																													3
2-5-11-7-0210			1																												1
2-5-13-8-0210				2																											2
2-5-14-8-0210					1																										1
2-5-15-8-0210						5																									5
2-5-16-8-0210							1																								1
2-6-7-9-0210								2																							2
2-6-9-9-0210									23	1^+^	1^+^																				25
2-6-18-9-0210										0																					0
2-6-NA-9-0210											0																				0
2-7-14-6-0210												0																			0
2-7-15-6-0210												1^+^	1																		2
2-7-9-9-0210														1																	1
2-8-10-8-0210						1*									0																1
2-8-11-8-0210																0							1^+^								1
2-8-11-NA-0210							1*										0														1
2-8-12-8-0210																		2													2
2-8-12-9-0210																			1												1
2-8-14-8-0210																				1											1
2-8-14-9-0210																					1										1
2-9-11-7-0210																						1									1
2-9-11-8-0210																							0								0
2-9-12-7-0210																								2							2
2-NA-9-7-0210																									5						5
3-7-13-8-0210																										0	1^+^				1
3-7-14-8-0210																											0				0
3-NA-10-7-0210																		1*										1			2
3-NA-12-7-0210								1*																					1		2
3-NA-7-7-0210																														1	1
**Total**	0	3	1	2	1	7	2	3	23	1	1	1	1	1	0	0	0	3	1	1	1	1	1	2	5	0	1	1	1	1	66

The grey shaded cells represent concordance of MLVA serotype of paired blood and stool samples. Data represent numbers.

^+^NTS pairs with MLVA types with variation in 1 of the rapidly changing loci (STTR5, STTR6 and STTR10) and no variation in the stable loci (STTR3 or STTR9): these are considered as identical isolates

*Typhimurium pairs with MLVA types with variation in > 1 of the rapidly changing loci (STTR5, STTR6 and STTR10) or variation in the stable loci (STTR3 or STTR9): these are considered as different isolates. Abbreviations: MLVA = multiple-locus variable-number of tandem repeats analysis, STTR = *Salmonella* Typhimurium tandem repeat.

**Table 4:** Overview of MLVA types of *Salmonella* Enteritidis paired isolates recovered from stool versus blood.

	Enteritidis MLVA types from blood						
Enteritidis MLVA types from stool	2-12-3-3-NA	2-13-3-3-NA	2-14-3-3-NA	2-15-3-3-NA	2-17-3-3-NA	2-18-3-3-NA	Total
2-12-3-3-NA	2						2
2-13-3-3-NA		4					4
2-14-3-3-NA			1				1
2-15-3-3-NA	1^+^			7			8
2-17-3-3-NA					3		3
2-18-3-3-NA						3	3
**Total**	3	4	1	7	3	3	21

The grey shaded cells represent concordance of MLVA serotype of paired blood and stool samples. Data represent numbers.

^+^Enteritidis pair with MLVA types with variation in one of the five loci: these are considered as identical isolates. Abbreviations: MLVA = multiple-locus variable-number of tandem repeats analysis.

**Fig 1:**
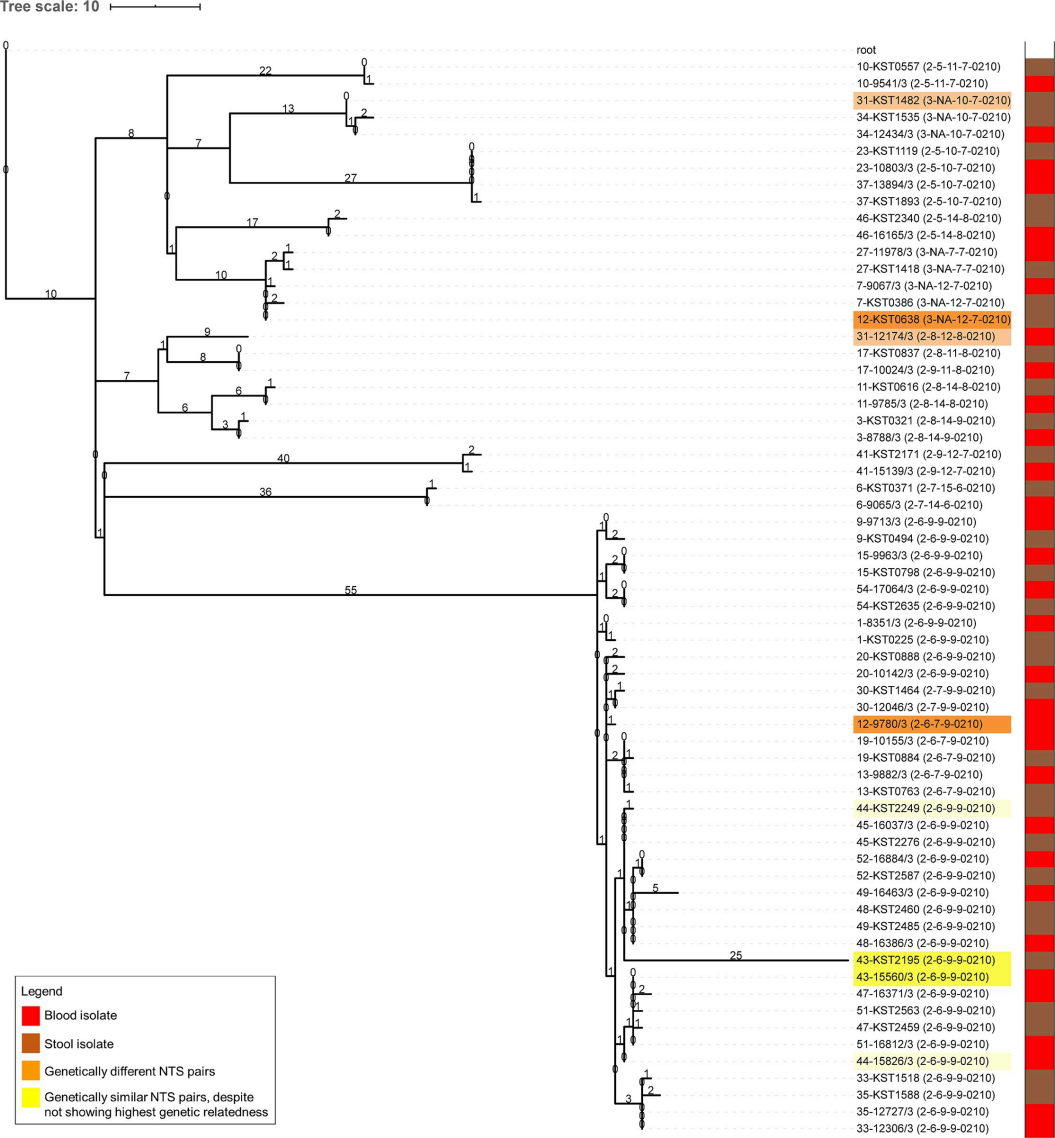
Phylogenetic tree of *Salmonella* Typhimurium pairs. Maximum likelihood phylogenetic tree of *Salmonella* Typhimurium paired isolates, mapped on reference strain *Salmonella* Typhimurium D23580 (Accession Number = NC_016854) and rooted on *Salmonella* Typhi 10040_15 (Accession Number = ERS1574281). The tree scale represents the number of SNPs, which are also annotated on the branches. The isolate names have a prefix representing the pair number followed by the study number of each isolate (S2 Table) and a suffix between brackets indicating the MVLA type. Isolates highlighted in orange are pairs showing low genetic similarity and were considered genetically different (orange highlight, pairs N° 12 and 31); isolates highlighted in yellow are pairs that despite not showing highest genetic relatedness were considered genetically similar based on the MLVA type, the SNP difference and the bootstrap values of the branch nodes (yellow highlight, pairs N° 43 and 44). The bar on the right indicates the specimen of isolation (blood isolate (red) versus stool isolate (brown)).

**Fig 2:**
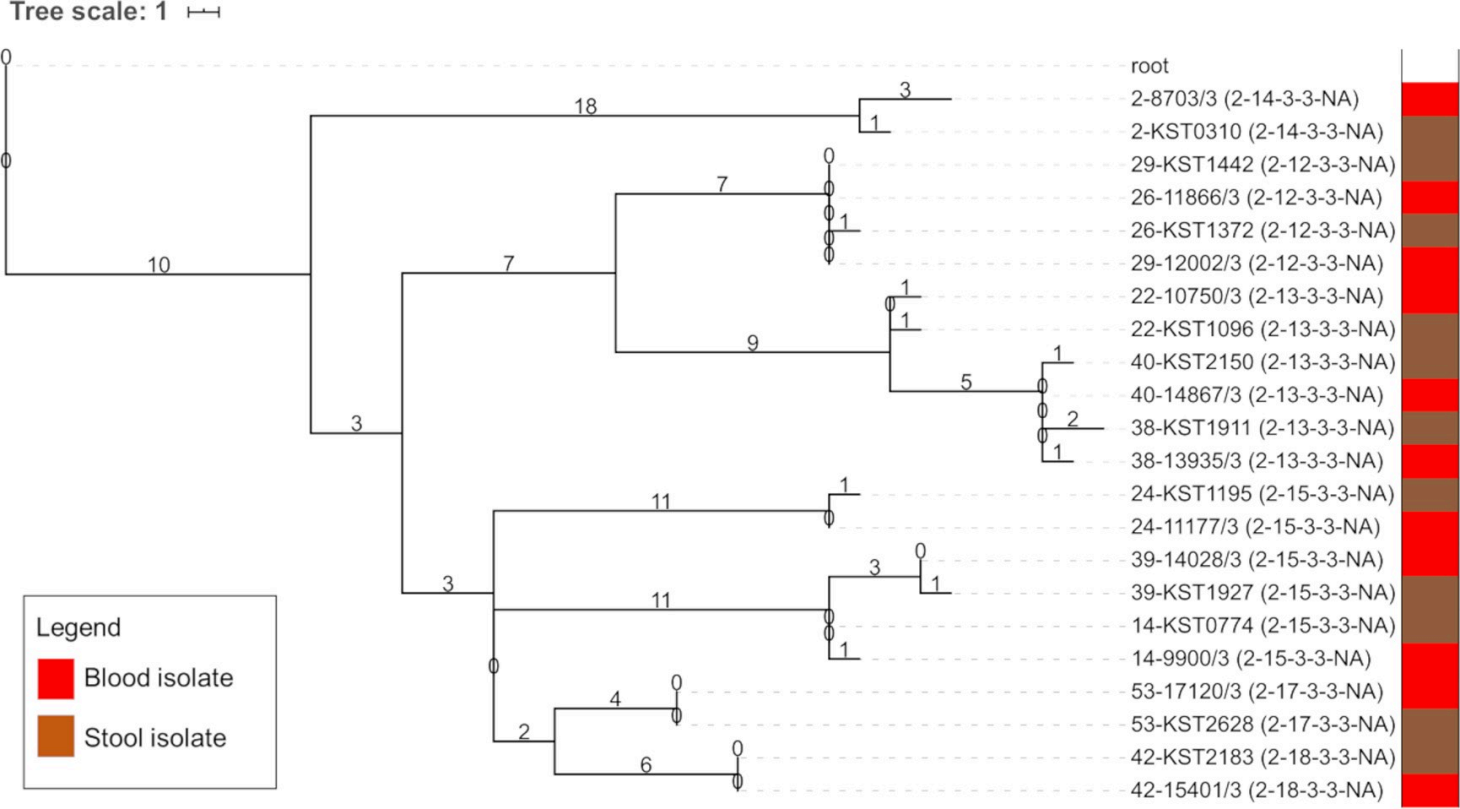
Phylogenetic tree of *Salmonella* Enteritidis pairs. Maximum likelihood phylogenetic tree of *Salmonella* Enteritidis paired isolates, mapped on reference strain *Salmonella* Enteritidis P125109 (Accession Number = AM933172) and rooted on *Salmonella* Typhi 10040_15 (Accession Number = ERS1574281). The tree scale represents the number of SNPs, which are also annotated on the branches. The isolate names have a prefix representing the pair number followed by the study number of each isolate (S2 Table) and a suffix between brackets indicating the MVLA type. The bar on the right indicates the specimen of isolation (blood isolate (red) versus stool isolate (brown)).

A genomics analysis was done on a subset of 43 NTS pairs consisting of 32/66 Typhimurium and 11/21 Enteritidis pairs. All analyzed Typhimurium isolates belonged to the ST313 sequence type lineage II and II.1 [8,33], while all Enteritidis isolates belonged to ST11 (S2 Table). A phylogenetic analysis (Figs 1 and 2) showed high genetic similarity for 30/32 (93.8%) Typhimurium and all Enteritidis pairs (11/11, 100%). The median number of SNP difference among NTS pairs was 1 (range 0–77) for Typhimurium pairs and 1 (range 0–4) for Enteritidis pairs. The Typhimurium pair with a delay between blood and stool sampling of 43 days had a difference of 1 SNP.

Agreement between MLVA and WGS was nearly perfect, with 39 Typhimurium and Enteritidis pairs showing identical MLVA types and high genetic similarity in the phylogenetic tree and two Typhimurium pairs showing different MLVA types and low genetic similarity. The latter pairs showed a difference of 45 and 77 SNPs and were considered genetically different (Fig 1, pairs highlighted in orange). In contrast, two Typhimurium pairs showed identical MLVA types but were not the most closely related isolates (Fig 1, pairs highlighted in yellow), with a difference of 3 and 28 SNPs respectively. For the latter pair, the stool isolate (sampled 6 days after blood) accumulated 25 of the 28 SNPs independently. As the genomic differences were small and the branch bifurcations lacked a full bootstrap support for these pairs (around 60%; S1 Fig); we considered both pairs genetically similar.

## Discussion

The present study demonstrated that nearly 30% of children with NTS BSI had stool excretion of a NTS isolate with high genetic similarity to the corresponding blood culture isolate. In contrast, NTS stool excretion was observed in only 2.1% of children admitted to the hospital without suspicion of BSI. Among the NTS blood and stool culture pairs, *Salmonella* Typhimurium and *Salmonella* Enteritidis belonged to ST313 lineage II and ST11 respectively.

Unlike for *Salmonella* Typhi the role of carriers in the transmission of NTS is unclear. NTS stool excretion has mainly been studied among patients recovering from NTS gastroenteritis in high-income countries [34,35]: median duration of stool excretion in children ≤ 5 years old is 7 weeks, with 2.6% excreting beyond a 1 year period [35]. In older children, durations are shorter (median 3–4 weeks). Furthermore, the duration of stool excretion is shorter for Typhimurium and antibiotic treatment may prolong excretion [34–36]. Even less is known about stool excretion of invasive NTS isolates in sSA. Most studies surveyed asymptomatic community members or food handlers [37,38]. Cross-sectional community-based studies showed 1.0% and 2.4% of NTS stool excretion among healthy persons in Senegal and Guinea-Bissau respectively [37]. Likewise, a recent community study in rural Kongo-Central in DR Congo showed 3.4% of NTS stool excretion (two consecutive stool samples) [39], which was in line with the proportion in the present control group (2.1%, single stool sample).

The high rate of NTS pairs (29.1% but probably higher given the single stool sampling under antibiotic coverage) provides incremental evidence for a human reservoir of invasive NTS. It complements studies showing NTS stool excretion among household members of index patients with NTS BSI [12,40]. In addition, MLVA types of stool isolates from the control group were similar to those of the NTS pairs from the NTS BSI group, which is in line with previous findings from the same area in DR Congo [39]. However, depending on the concentration of NTS excreted in the stool (which is currently not known), growth of NTS in contaminated food or water may be needed to reach the infectious dose as is the case in high-resource settings [13]. Further, the current evidence on transmission of NTS does not exclude animals as a reservoir of NTS [41].

The finding of NTS pairs may also shed a light on the pathophysiology of NTS infections. The stool excretion of NTS can be interpreted as intestinal infection from the onset of symptoms but might also represent intestinal colonization with bacterial translocation from the gut and subsequent systemic dissemination. This "typhoid fever-like" scenario would be in line with the clinical presentation of invasive NTS infections in sSA, *i*.*e*. febrile systemic fever with non-specific clinical features, without gastroenteritis [13] but with increased gut permeability driven by *Plasmodium falciparum* sequestration and altered gut microbiota associated with malnutrition [42,43].

The cross-sectional study design had limitations. Children and their parents were addressed for enrollment only after blood culture growth. As a consequence, there was a delay between blood and stool sampling and stool sampling was done under antibiotic treatment. In addition, the study was set up in a capacity building program and relied on regular clinical staff for participant recruitment during working hours which meant that only a few patients per day were included. Likewise, the control group was selected by convenience. We do not believe that this had a significant impact on the results, as the control group was large (1598 children), thereby ensuring a wide variety of clinical presentations. Due to the limited diagnostic platforms available, it was not possible to make a reliable clinical diagnosis for the individual control patients, however, the most common diagnoses of children admitted for non-febrile diseases during the study period were known (see results). Collecting stool samples in small and often severely ill children proved to be difficult. Only a single stool sample was collected while it is known that sampling on consecutive days increases yields [44]. Lastly, the date of onset of symptoms was not registered, nor was the occurrence of gastrointestinal symptoms. As to its strengths, blood cultures were systematically sampled based on harmonized indications and the hospital staff got the trust of the patient's families facilitating enrollment [14,15].

Future research is needed to put the present findings into perspective. A prospective study with blood and stool cultures directly after onset of symptoms and with consecutive sampling during convalescence could clarify onset and duration of NTS stool excretion. Assessing the concentration of NTS in stool samples could give an idea about its infectivity (*e*.*g*. 10^6^ to 10^7^ NTS organisms per gram of feces in case of NTS gastroenteritis) [34]. A difficult point will be the assessment of the infectious dose, as human challenge experiments typically address adult patients whereas children have the highest risk for NTS BSI [42,45]. As to the aforementioned studies, WGS should cover the diversity of infecting organisms within a particular specimen and time-point, *i*.*e*. by assessing multiple isolates per sample [46,47].

In conclusion, the present study, conducted in an endemic area for NTS showed that nearly 30% of children with NTS bloodstream infection had stool excretion of an NTS isolate with high genetic similarity. This observation adds to the evidence of humans as a potential reservoir for NTS.

## Supporting information

S1 Checklist STROBE checklist. Checklist of items that should be included in reports of observational studies.
(DOC)

S1 Table Proportions and percentages of stool sampling done in the NTS bloodstream infection group, per month and per year. The rainy season at the Kisantu sampling site comprises the months October to May, the dry season comprises the months June to September. Abbreviations: NTS = non-typhoidal *Salmonella*.
(DOCX)

S2 Table Overview of the non-typhoidal *Salmonella* pairs. Abbreviations: MLVA = multiple-locus variable-number of tandem repeats analysis, MLST = multi-locus sequence type, ND = no data, NA = not applicable, SNP = single nucleotide polymorphism, WGS = whole genome sequencing.
(DOCX)

S3 Table Overview of MLVA types of NTS stool isolates recovered from the control group. Abbreviations: MLVA = multiple-locus variable-number of tandem repeats analysis, NA = not applicable, NTS = non-typhoidal *Salmonella*.
(DOCX)

S1 Fig Phylogenetic tree of *Salmonella* Typhimurium pairs with bootstrap values of branch nodes. Maximum likelihood phylogenetic tree of *Salmonella* Typhimurium paired isolates, mapped on reference strain *Salmonella* Typhimurium D23580 (Accession Number = NC_016854) and rooted on *Salmonella* Typhi 10040_15 (Accession Number = ERS1574281). The bootstrap values of the branch nodes are indicated on the branches. The branch lengths represent the nucleotide substitution rate in the core SNP alignment of 58148 SNPs. The isolate names have a prefix representing the pair number followed by the study number of each isolate (S2 Table) and a suffix between brackets indicating the MVLA type. The bar on the right indicates the specimen of isolation (blood isolate (red) versus stool isolate (brown)).
(TIF)
