# Supplementary material for: High genetic similarity between non-typhoidal Salmonella isolated from paired blood and stool samples of children in the Democratic Republic of the Congo
Source: PLoS Negl Trop Dis. 2020 Jul 2;14(7):e0008377. doi: 10.1371/journal.pntd.0008377 (PMC7331982; doi:10.1371/journal.pntd.0008377)
Supplement: S1 Checklist — Checklist of items that should be included in reports of observational studies. (DOC) [file pntd.0008377.s001.doc]

STROBE Statement—Checklist of items that should be included in reports of ***cross-sectional studies***

**Manuscript:** **“High genetic similarity between non-typhoidal *Salmonella* isolated from paired blood and stool samples of children in the Democratic Republic of the Congo.”**

|  | Item No | Recommendation |
| --- | --- | --- |
| **Title and abstract** | 1 | (*a*) Indicate the study’s design with a commonly used term in the title or the abstract  **Line 57**: The methods section mentions the “cross-sectional study” design. |
|  |  | (*b*) Provide in the abstract an informative and balanced summary of what was done and what was found  **Lines 56-61 (Abstract methods section) describes what was done:**  Between November 2013 and April 2017, hospital-admitted children (29 days to 14 years) with culture-confirmed NTS bloodstream infections were enrolled in a cross-sectional study at Kisantu Hospital, DR Congo. Stool cultures for Salmonella were performed on a subset of enrolled children, as well as on a control group of non-febrile hospital-admitted children. Pairs of blood and stool NTS isolates were assessed for genetic similarity by multiple-locus variable-number of tandem repeats (MLVA) and genomics analysis.  **Lines 63-72 (Abstract results section) describes what was found:**  A total of 299 children with NTS grown from blood cultures (Typhimurium 68.6%, Enteritidis 30.4%, other NTS 1.0%) had a stool sample processed; in 105 (35.1%) of them NTS was detected (Typhimurium 70.5%, Enteritidis 25.7%, other NTS 3.8%). A total of 87/105 (82.9%) pairs of blood and stool NTS isolates were recovered (representing 29.1% of the 299 children). Among 1598 controls, the proportion of NTS stool excretion was 2.1% (p < 0.0001). MLVA types among paired isolates were identical in 82/87 (94.3%) pairs (61/66 (92.4%) in Typhimurium and 21/21 (100%) in Enteritidis pairs). Genomics analysis confirmed high genetic similarity within 41/43 (95.3%) pairs, showing a median SNP difference of 1 (range 0 - 77) and 1 (range 0 - 4) for Typhimurium and Enteritidis pairs respectively. Typhimurium and Enteritidis isolates belonged to sequence types ST313 lineage II and ST11 respectively. |
| Introduction | | |
| Background/rationale | 2 | Explain the scientific background and rationale for the investigation being reported  **Lines 91-98 explain the scientific background for the investigation being reported:**  Non-typhoidal Salmonella (NTS) is a leading cause of bloodstream infections (BSI) with an estimated global burden of invasive NTS infections of 535,000 cases and a case fatality rate of 14.5% in 2017. The burden is highest in sub-Saharan Africa (sSA) and in children under five years of age [1]. Salmonella enterica subspecies enterica serotypes Typhimurium and Enteritidis are the most common causes of NTS BSI [2–5]. The pathogenicity of NTS differs between high-income countries and sSA with a higher risk of invasive disease and associated mortality in the latter [6]; this can be partly explained by the emergence of distinct and invasive clades of Typhimurium and Enteritidis serotypes across sSA [7–10].  **Lines 98-102 explain the rationale for the investigation being reported:**  Unlike Salmonella Typhi for which the role of human carriers is pivotal in the transmission [3,11], little is known about the reservoirs and transmission of NTS in sSA, but environmental, zoonotic and human sources have been hypothesized [3,12,13]. The role of NTS excretion in the stool has not yet been elucidated and it is unknown whether the NTS strains in the blood of BSI patients are also excreted in stool [3,13]. |
| Objectives | 3 | State specific objectives, including any prespecified hypotheses  **Lines 104-107 state the specific objectives:**  The objectives of this study were to determine the proportion of NTS stool excretion among hospital-admitted children with NTS BSI and non-febrile hospitalized children as a control group, and to assess the genetic similarity of paired blood and stool NTS isolates (i.e. identical NTS serotypes obtained from the same patient in the course of the hospital admission). |
| Methods | | |
| Study design | 4 | Present key elements of study design early in the paper  **Lines 119-126 present key elements of the study design:**  A cross-sectional study in which hospital-admitted patients aged 29 days to 14 years old with culture-confirmed NTS BSI were enrolled (further referred to as “NTS BSI group”). Enrollment depended on the availability of regular clinical staff during office hours. As soon as possible after enrollment, a stool sample was collected for culture. Stool cultures were also sampled in a control group selected by convenience and consisting of non-febrile hospital-admitted children, as they were presumed to be most closely related to the NTS BSI group in terms of demographics and residency or referring health center. A single stool culture was sampled per patient – repeat cultures were removed from analysis and only the first NTS isolate was considered. |
| Setting | 5 | Describe the setting, locations, and relevant dates, including periods of recruitment, exposure, follow-up, and data collection  **Lines 111-116 describe the study site and study period:**  The study took place from November 2013 to April 2017 at the hospital of Saint-Luc in Kisantu (HSLK), Kongo-Central province, Democratic Republic of the Congo (DR Congo). HSLK is the major sampling site of the microbiological surveillance network organized by the Institut National de Recherche Biomédicale (INRB), Kinshasa, DR Congo, and the Institute of Tropical Medicine, Antwerp, Belgium [4,14]. Over a 10-year period (2007 – 2017), NTS ranked first as a cause of BSI in children accounting for 63.8% of culture-confirmed BSI [14,15] |
| Participants | 6 | (*a*) Give the eligibility criteria, and the sources and methods of selection of participants  **Lines 119-121 explain the methods of selection and enrolment of participants:**  A cross-sectional study in which hospital-admitted patients aged 29 days to 14 years old with culture-confirmed NTS BSI were enrolled (further referred to as “NTS BSI group”). Enrollment depended on the availability of regular clinical staff during office hours. |
| Variables | 7 | Clearly define all outcomes, exposures, predictors, potential confounders, and effect modifiers. Give diagnostic criteria, if applicable  **Lines 128-140** describe the methods related to the processing of the blood and stool cultures for diagnosis of *Salmonella*.  **Lines 142-148** describe the MLVA typing, definitions and reporting.  **Lines 150-170** describe the methods and definitions related to the whole genome sequencing and maximum likelihood phylogenetic tree analysis.  **Lines 172-180** describe the definitions according to diagnostic criteria that were used in the manuscript. |
| Data sources/ measurement | 8* | For each variable of interest, give sources of data and details of methods of assessment (measurement). Describe comparability of assessment methods if there is more than one group  **Lines 183-187 describe the data collection and analysis used to assess the data:**  Data were encoded into an Excel database (Microsoft, Redmond, Washington). Data were characterized by proportions, percentages, ratios, medians, 25-75% interquartile ranges (IQR) and ranges. Differences between proportions were tested for significance using the χ2 test or the McNemar’s test in case of correlated proportions; medians were compared using the Mann-Whitney U test. A p-value of < 0.05 was considered significant.  **Lines 128-170 describe the phenotypical and genotypical (MLVA, Whole Genome Sequencing) data analysis.** See above (Variables). |
| Bias | 9 | Describe any efforts to address potential sources of bias  - **A control group** of children was assessed for comparison (lines 122-125) and composition of control group was assessed and compared to the NTS BSI group (lines 204-209).  - **Patient inclusion** was consistent over the study period (lines 209-210).  - **Proportions** of serotype distributions were assessed for each patient group (NTS BSI group vs NTS BSI group with stool culture taken, NTS BSI group with NTS stool excretion vs control group), see Table 1 and lines 220-227.  - **Rigid measurements and definitions of genotypic similarity** as accepted for epidemic analysis (maximum likelihood phylogenetic tree, MLVA types) were used to assess and express genetic similarity (lines 173-180).  - **Lines 352-366: Potential limitations inherent to the study design and settings have been mentioned and their potential impact has been measured and discussed**: (i) delay between blood and stool culture sampling (data provided, lines 234-239), (ii) inclusion according to availability of staff, (iii) sampling of a single stool sample, (iv) lack of data about date of onset of symptoms and occurrence of gastrointestinal symptoms. |
| Study size | 10 | Explain how the study size was arrived at  In view of the explorative nature of the study, the study protocol did not include a sample size calculation (as the proportions of stool culture positivity were not known), but mentioned to include 2000 patients. The total number of 1897 enrolled children (299 children with NTS BSI and 1598 non-febrile children in the control group) and a total number of 87 pairs of blood and stool isolates, was sufficient to allow us to indicate genetic similarity for both Typhimurium and Enteritidis serotypes (see Table 1 and lines 220-227). |
| Quantitative variables | 11 | Explain how quantitative variables were handled in the analyses. If applicable, describe which groupings were chosen and why  **Lines 183-187 describe the data collection and analysis used to assess the data:**  Data were encoded into an Excel database (Microsoft, Redmond, Washington). Data were characterized by proportions, percentages, ratios, medians, 25-75% interquartile ranges (IQR) and ranges. Differences between proportions were tested for significance using the χ2 test or the McNemar’s test in case of correlated proportions; medians were compared using the Mann-Whitney U test. A p-value of < 0.05 was considered significant. |
| Statistical methods | 12 | (*a*) Describe all statistical methods, including those used to control for confounding  **Lines 183-187 describe the statistical analysis used to assess the data:**  Data were characterized by proportions, percentages, ratios, medians, 25-75% interquartile ranges (IQR) and ranges. Differences between proportions were tested for significance using the χ2 test or the McNemar’s test in case of correlated proportions; medians were compared using the Mann-Whitney U test. A p-value of < 0.05 was considered significant. |
|  |  | (*b*) Describe any methods used to examine subgroups and interactions  **Not applicable** |
|  |  | (*c*) Explain how missing data were addressed  **Not applicable** |
|  |  | (*d*) If applicable, describe analytical methods taking account of sampling strategy  **Not applicable** |
|  |  | (*e*) Describe any sensitivity analyses  **Not applicable** |
| Results | | |
| Participants | 13* | (a) Report numbers of individuals at each stage of study—eg numbers potentially eligible, examined for eligibility, confirmed eligible, included in the study, completing follow-up, and analysed  The study was explorative and assessed pairs of stool/blood culture samples within index patients with NTS bloodstream infection, sampled and identified during routine patient care. Given the embedment in clinical care (selection and inclusion of patients) and the practical difficulties of sampling (delay of growth of blood cultures, small children), registration of eligible and consenting patients was not systematically done. In addition, part of the parents consented but later on did not provide a sample for reasons of death (n = 493 over the entire study period), leaving the hospital (for financial reasons) against medical advice (n = 58) or omission to sample due to the stressful situation of hospital admission – these events were neither registered. Overall, the subset of children who provided a stool sample was 299/1052 (28.4%) and was consistent over time. (Table 1 and S1 Table).  **Line 202:** Among 1052 children with NTS grown from blood cultures, 299 (28.4%) had a stool sample processed (Table 1).  **Lines 209-210:** Patient inclusion was consistent over the study period, varying between 19.8% (2014) and 36.8% (2013) respectively (S1 Table). |
|  |  | (b) Give reasons for non-participation at each stage  Reasons for non-participation at each stage were not registered. |
|  |  | (c) Consider use of a flow diagram |
| Descriptive data | 14* | (a) Give characteristics of study participants (eg demographic, clinical, social) and information on exposures and potential confounders  **Lines 203-209:**  Their median age (available for 298/299 (99.7%) children) was 1.5 (IQR 0.9-2.6) years; 78.2% (233/298) were < 36 months and 92.3% (275/298) were < 5 years old. The control group consisted of 1598 children; their median age (available for 100% of children) was 1.8 (IQR 0.9-3.8) years. The median age between both groups did not differ significantly (p = 0.02) and the male-to-female ratio was identical (1:0.9). Overall, the most common diagnoses (n=4349) of children admitted for non-febrile diseases during the study period were sickle cell anemia (31.6%), malnutrition (23.4%) and amoebiasis (6.4%). |
|  |  | (b) Indicate number of participants with missing data for each variable of interest  **Lines 202-206:**  Their median age (available for 298/299 (99.7%) children) was 1.5 (IQR 0.9-2.6) years; 78.2% (233/298) were < 36 months and 92.3% (275/298) were < 5 years old. The control group consisted of 1598 children; their median age (available for 100% of children) was 1.8 (IQR 0.9-3.8) years. |
| Outcome data | 15* | Report numbers of outcome events or summary measures  **Non applicable** |
| Main results | 16 | (*a*) Give unadjusted estimates and, if applicable, confounder-adjusted estimates and their precision (eg, 95% confidence interval). Make clear which confounders were adjusted for and why they were included  **Lines 220-227** and Table 1 describe the proportions and the serotype distribution of the grown cultures.  **Lines 229-239**, Table 2 and S2 Table describe the NTS pairs from blood and stool (serotype distribution, proportions, median delay between blood and stool sampling).  **Lines 249-254**, **lines 273-279, lines 303-312**, Tables 3 and 4, S2 Table, S3 Table, Figures 1 and 2 and S1 Figure describe the MLVA and genomics analysis and the genetic similarity within NTS pairs. |
|  |  | (*b*) Report category boundaries when continuous variables were categorized  **Not applicable** |
|  |  | (*c*) If relevant, consider translating estimates of relative risk into absolute risk for a meaningful time period  **Not applicable** |
| Other analyses | 17 | Report other analyses done—eg analyses of subgroups and interactions, and sensitivity analyses  **Not applicable** |
| Discussion | | |
| Key results | 18 | Summarise key results with reference to study objectives  **Lines 315-319 summarise the key results:**  The present study demonstrated that nearly 30% of children with NTS BSI had stool excretion of a NTS isolate with high genetic similarity to the corresponding blood culture isolate. In contrast, NTS stool excretion was observed in only 2.1% of children admitted to the hospital without suspicion of BSI. Among the NTS blood and stool culture pairs, Salmonella Typhimurium and Salmonella Enteritidis belonged to ST313 lineage II and ST11 respectively. |
| Limitations | 19 | Discuss limitations of the study, taking into account sources of potential bias or imprecision. Discuss both direction and magnitude of any potential bias  **Lines 352-366 discuss the limitations of the study:**  The cross-sectional study design had limitations. Children and their parents were addressed for enrollment only after blood culture growth. As a consequence, there was a delay between blood and stool sampling and stool sampling was done under antibiotic treatment. In addition, the study was set up in a capacity building program and relied on regular clinical staff for participant recruitment during working hours which meant that only a few patients per day were included. Likewise, the control group was selected by convenience. We do not believe that this had a significant impact on the results, as the control group was large (1598 children), thereby ensuring a wide variety of clinical presentations. Due to the limited diagnostic platforms available, it was not possible to make a reliable clinical diagnosis for the individual control patients, however, the most common diagnoses of children admitted for non-febrile diseases during the study period were known (see results). Collecting stool samples in small and often severely ill children proved to be difficult. Only a single stool sample was collected while it is known that sampling on consecutive days increases yields [44]. Lastly, the date of onset of symptoms was not registered, nor was the occurrence of gastrointestinal symptoms. As to its strengths, blood cultures were systematically sampled based on harmonized indications and the hospital staff got the trust of the patient's families facilitating enrollment [14,15]. |
| Interpretation | 20 | Give a cautious overall interpretation of results considering objectives, limitations, multiplicity of analyses, results from similar studies, and other relevant evidence  **Lines 321-350 give an interpretation of the results.** |
| Generalisability | 21 | Discuss the generalisability (external validity) of the study results  **Lines 368-376 discuss the need for future research to put the present findings into perspective and list the conclusions.** |
| Other information | | |
| Funding | 22 | Give the source of funding and the role of the funders for the present study and, if applicable, for the original study on which the present article is based  This work was funded by the Belgian Directorate of Development Cooperation (DGD) through Project 2.01 of the Third Framework Agreement between the Belgian DGD and the Institute of Tropical Medicine, Belgium, and by the Baillet-Latour fund. |

*Give information separately for exposed and unexposed groups.

**Note:** An Explanation and Elaboration article discusses each checklist item and gives methodological background and published examples of transparent reporting. The STROBE checklist is best used in conjunction with this article (freely available on the Web sites of PLoS Medicine at http://www.plosmedicine.org/, Annals of Internal Medicine at http://www.annals.org/, and Epidemiology at http://www.epidem.com/). Information on the STROBE Initiative is available at www.strobe-statement.org.
