# Supplementary material for: High genetic similarity between non-typhoidal Salmonella isolated from paired blood and stool samples of children in the Democratic Republic of the Congo
Source: PLoS Negl Trop Dis. 2020 Jul 2;14(7):e0008377. doi: 10.1371/journal.pntd.0008377 (PMC7331982; doi:10.1371/journal.pntd.0008377)
Supplement: S1 Table — The rainy season at the Kisantu sampling site comprises the months October to May, the dry season comprises the months June to September. Abbreviations: NTS = non-typhoidal Salmonella. (DOCX) [file pntd.0008377.s002.docx]

**S1 Table. Proportions and percentages of stool sampling done in the NTS bloodstream infection group, per month and per year.**

|  | **2013** | **2014** | **2015** | **2016** | **2017** | **Total** |
| --- | --- | --- | --- | --- | --- | --- |
| January |  | 3/19 (15.8%) | 16/70 (22.9%) | 9/19 (47.4%) | 12/55 (21.8%) | 40/163 (24.5%) |
| February |  | 11/36 (30.6%) | 21/41 (51.2%) | 4/14 (28.6%) | 10/51 (19.6%) | 46/142 (32.4%) |
| March |  | 7/45 (15.6%) | 9/21 (42.9%) | 8/38 (21.1%) | 12/39 (30.8%) | 36/143 (25.2%) |
| April |  | 0/18 (0.0%) | 4/11 (36.4%) | 11/38 (28.9%) | 4/21 (19.0%) | 19/88 (21.6%) |
| May |  | 1/15 (6.7%) | 1/12 (8.3%) | 16/27 (59.3%) |  | 18/54 (33.3%) |
| June |  | 2/6 (33.3%) | 4/16 (25.0%) | 8/28 (28.6%) |  | 14/50 (28.0%) |
| July |  | 2/4 (50.0%) | 2/7 (28.6%) | 11/20 (55.0%) |  | 15/31 (48.4%) |
| August |  | 2/5 (40.0%) | 4/9 (44.4%) | 10/21 (47.6%) |  | 16/35 (45.7%) |
| September |  | 4/19 (21.1%) | 4/9 (44.4%) | 4/26 (15.4%) |  | 12/54 (22.2%) |
| October |  | 7/18 (38.9%) | 2/6 (33.3%) | 5/10 (50.0%) |  | 14/34 (41.2%) |
| November | 7/15 (46.7%) | 2/28 (7.1%) | 3/23 (13.0%) | 13/46 (28.3%) |  | 25/112 (22.3%) |
| December | 10/29 (34.5%) | 8/35 (22.9%) | 6/29 (20.7%) | 20/53 (37.7%) |  | 44/146 (30.1%) |
| **Total** | 17/44 (38.6%) | 49/248 (19.8%) | 76/254 (29.9%) | 119/340 (35.0%) | 38/166 (22.9%) | 299/1052 (28.4%) |

The rainy season at the Kisantu sampling site comprises the months October to May, the dry season comprises the months June to September. Abbreviations: NTS = non-typhoidal *Salmonella.*
