# Supplementary material for: High genetic similarity between non-typhoidal Salmonella isolated from paired blood and stool samples of children in the Democratic Republic of the Congo
Source: PLoS Negl Trop Dis. 2020 Jul 2;14(7):e0008377. doi: 10.1371/journal.pntd.0008377 (PMC7331982; doi:10.1371/journal.pntd.0008377)
Supplement: S2 Table — Abbreviations: MLVA = multiple-locus variable-number of tandem repeats analysis, MLST = multi-locus sequence type, ND = no data, NA = not applicable, SNP = single nucleotide polymorphism, WGS = whole genome sequencing. (DOCX) [file pntd.0008377.s003.docx]

**S2 Table. Overview of the non-typhoidal *Salmonella* pairs.**

| **Pair N°** | **Study N°** | **Specimen** | **Year of sampling** | **Delay stool sampling**  **(N days after blood sampling)** | **Serotype** | **MLVA type** | **Identical MLVA type** | **Selected for WGS** | **Accession N°** | **MLST type** | **Genetic similarity in tree** | **SNP difference** |
| --- | --- | --- | --- | --- | --- | --- | --- | --- | --- | --- | --- | --- |
| 1 | 8351/3 | Blood | 2014 | NA | *Salmonella* Typhimurium | 2-6-9-9-0210 | YES | YES | ERS4406557 | ST313 | YES | 1 |
|  | KST0225 | Stool | 2014 | 11 | *Salmonella* Typhimurium | 2-6-9-9-0210 |  | YES | ERS4406570 | ST313 |  |  |
| 2 | 8703/3 | Blood | 2014 | NA | *Salmonella* Enteritidis | 2-14-3-3-NA | YES | YES | ERS4406559 | ST11 | YES | 4 |
|  | KST0310 | Stool | 2014 | 3 | *Salmonella* Enteritidis | 2-14-3-3-NA |  | YES | ERS4406572 | ST11 |  |  |
| 3 | 8788/3 | Blood | 2014 | NA | *Salmonella* Typhimurium | 2-8-14-9-0210 | YES | YES | ERS4406560 | ST313 | YES | 1 |
|  | KST0321 | Stool | 2014 | 2 | *Salmonella* Typhimurium | 2-8-14-9-0210 |  | YES | ERS4406571 | ST313 |  |  |
| 4 | 8956/3 | Blood | 2014 | NA | *Salmonella* Typhimurium | 2-6-18-9-0210 | YES | NO | NA | ND | NA | NA |
|  | KST0348 | Stool | 2014 | 1 | *Salmonella* Typhimurium | 2-6-9-9-0210 |  | NO | NA | ND |  |  |
| 5 | 8966/3 | Blood | 2014 | NA | *Salmonella* Enteritidis | 2-17-3-3-NA | YES | NO | NA | ND | NA | NA |
|  | KST0352 | Stool | 2014 | 2 | *Salmonella* Enteritidis | 2-17-3-3-NA |  | NO | NA | ND |  |  |
| 6 | 9065/3 | Blood | 2014 | NA | *Salmonella* Typhimurium | 2-7-14-6-0210 | YES | YES | ERS4406561 | ST313 | YES | 1 |
|  | KST0371 | Stool | 2014 | 1 | *Salmonella* Typhimurium | 2-7-15-6-0210 |  | YES | ERS4406573 | ST313 |  |  |
| 7 | 9067/3 | Blood | 2014 | NA | *Salmonella* Typhimurium | 3-NA-12-7-0210 | YES | YES | ERS4406563 | ST313 | YES | 3 |
|  | KST0386 | Stool | 2014 | 8 | *Salmonella* Typhimurium | 3-NA-12-7-0210 |  | YES | ERS4406574 | ST313 |  |  |
| 8 | 9612/3 | Blood | 2014 | NA | *Salmonella* Typhimurium | 3-7-14-8-0210 | YES | NO | NA | ND | NA | NA |
|  | KST0444 | Stool | 2014 | 1 | *Salmonella* Typhimurium | 3-7-13-8-0210 |  | NO | NA | ND |  |  |
| 9 | 9713/3 | Blood | 2014 | NA | *Salmonella* Typhimurium | 2-6-9-9-0210 | YES | YES | ERS4406564 | ST313 | YES | 2 |
|  | KST0494 | Stool | 2014 | 0 | *Salmonella* Typhimurium | 2-6-9-9-0210 |  | YES | ERS4406575 | ST313 |  |  |
| 10 | 9541/3 | Blood | 2014 | NA | *Salmonella* Typhimurium | 2-5-11-7-0210 | YES | YES | ERS4406562 | ST313 | YES | 1 |
|  | KST0557 | Stool | 2014 | 43 | *Salmonella* Typhimurium | 2-5-11-7-0210 |  | YES | ERS4406576 | ST313 |  |  |
| 11 | 9785/3 | Blood | 2014 | NA | *Salmonella* Typhimurium | 2-8-14-8-0210 | YES | YES | ERS4406566 | ST313 | YES | 1 |
|  | KST0616 | Stool | 2014 | -7 | *Salmonella* Typhimurium | 2-8-14-8-0210 |  | YES | ERS4406577 | ST313 |  |  |
| 12 | 9780/3 | Blood | 2014 | NA | *Salmonella* Typhimurium | 2-6-7-9-0210 | NO | YES | ERS4406565 | ST313 | NO | 77 |
|  | KST0638 | Stool | 2014 | 1 | *Salmonella* Typhimurium | 3-NA-12-7-0210 |  | YES | ERS4406578 | ST313 |  |  |
| 13 | 9882/3 | Blood | 2014 | NA | *Salmonella* Typhimurium | 2-6-7-9-0210 | YES | YES | ERS4406567 | ST313 | YES | 1 |
|  | KST0763 | Stool | 2014 | 1 | *Salmonella* Typhimurium | 2-6-7-9-0210 |  | YES | ERS4406579 | ST313 |  |  |
| 14 | 9900/3 | Blood | 2014 | NA | *Salmonella* Enteritidis | 2-15-3-3-NA | YES | YES | ERS4406568 | ST11 | YES | 1 |
|  | KST0774 | Stool | 2014 | 1 | *Salmonella* Enteritidis | 2-15-3-3-NA |  | YES | ERS4406580 | ST11 |  |  |
| 15 | 9963/3 | Blood | 2014 | NA | *Salmonella* Typhimurium | 2-6-9-9-0210 | YES | YES | ERS4406569 | ST313 | YES | 0 |
|  | KST0798 | Stool | 2014 | 0 | *Salmonella* Typhimurium | 2-6-9-9-0210 |  | YES | ERS4406584 | ST313 |  |  |
| 16 | 10002/3 | Blood | 2014 | NA | *Salmonella* Enteritidis | 2-18-3-3-NA | YES | NO | NA | ND | NA | NA |
|  | KST0820 | Stool | 2014 | 0 | *Salmonella* Enteritidis | 2-18-3-3-NA |  | NO | NA | ND |  |  |
| 17 | 10024/3 | Blood | 2014 | NA | *Salmonella* Typhimurium | 2-9-11-8-0210 | YES | YES | ERS4406531 | ST313 | YES | 0 |
|  | KST0837 | Stool | 2014 | 2 | *Salmonella* Typhimurium | 2-8-11-8-0210 |  | YES | ERS4406585 | ST313 |  |  |
| 18 | 10100/3 | Blood | 2014 | NA | *Salmonella* Typhimurium | 2-6-NA-9-0210 | YES | NO | NA | ND | NA | NA |
|  | KST0865 | Stool | 2014 | 1 | *Salmonella* Typhimurium | 2-6-9-9-0210 |  | NO | NA | ND |  |  |
| 19 | 10155/3 | Blood | 2014 | NA | *Salmonella* Typhimurium | 2-6-7-9-0210 | YES | YES | ERS4406581 | ST313 | YES | 1 |
|  | KST0884 | Stool | 2014 | 2 | *Salmonella* Typhimurium | 2-6-7-9-0210 |  | YES | ERS4406586 | ST313 |  |  |
| 20 | 10142/3 | Blood | 2014 | NA | *Salmonella* Typhimurium | 2-6-9-9-0210 | YES | YES | ERS4406530 | ST313 | YES | 4 |
|  | KST0888 | Stool | 2014 | 1 | *Salmonella* Typhimurium | 2-6-9-9-0210 |  | YES | ERS4406587 | ST313 |  |  |
| 21 | 10393/3 | Blood | 2014 | NA | *Salmonella* Typhimurium | 2-6-9-9-0210 | YES | NO | NA | ND | NA | NA |
|  | KST1002 | Stool | 2014 | 3 | *Salmonella* Typhimurium | 2-6-9-9-0210 |  | NO | NA | ND |  |  |
| 22 | 10750/3 | Blood | 2014 | NA | *Salmonella* Enteritidis | 2-13-3-3-NA | YES | YES | ERS4406582 | ST11 | YES | 2 |
|  | KST1096 | Stool | 2014 | 2 | *Salmonella* Enteritidis | 2-13-3-3-NA |  | YES | ERS4406589 | ST11 |  |  |
| 23 | 10803/3 | Blood | 2014 | NA | *Salmonella* Typhimurium | 2-5-10-7-0210 | YES | YES | ERS4406583 | ST313 | YES | 0 |
|  | KST1119 | Stool | 2014 | 7 | *Salmonella* Typhimurium | 2-5-10-7-0210 |  | YES | ERS4406588 | ST313 |  |  |
| 24 | 11177/3 | Blood | 2014 | NA | Salmonella Enteritidis | 2-15-3-3-NA | YES | YES | ERS4406532 | ST11 | YES | 1 |
|  | KST1195 | Stool | 2014 | 1 | Salmonella Enteritidis | 2-15-3-3-NA |  | YES | ERS4406590 | ST11 |  |  |
| 25 | 11874/3 | Blood | 2015 | NA | *Salmonella* Enteritidis | 2-12-3-3-NA | YES | NO | NA | ND | NA | NA |
|  | KST1371 | Stool | 2015 | 0 | *Salmonella* Enteritidis | 2-15-3-3-NA |  | NO | NA | ND |  |  |
| 26 | 11866/3 | Blood | 2015 | NA | *Salmonella* Enteritidis | 2-12-3-3-NA | YES | YES | ERS4406534 | ST11 | YES | 1 |
|  | KST1372 | Stool | 2015 | 0 | *Salmonella* Enteritidis | 2-12-3-3-NA |  | YES | ERS4406592 | ST11 |  |  |
| 27 | 11978/3 | Blood | 2015 | NA | *Salmonella* Typhimurium | 3-NA-7-7-0210 | YES | YES | ERS4406533 | ST313 | YES | 2 |
|  | KST1418 | Stool | 2015 | 0 | *Salmonella* Typhimurium | 3-NA-7-7-0210 |  | YES | ERS4406593 | ST313 |  |  |
| 28 | 11968/3 | Blood | 2015 | NA | *Salmonella* Enteritidis | 2-15-3-3-NA | YES | NO | NA | ND | NA | NA |
|  | KST1441 | Stool | 2015 | 2 | *Salmonella* Enteritidis | 2-15-3-3-NA |  | NO | NA | ND |  |  |
| 29 | 12002/3 | Blood | 2015 | NA | *Salmonella* Enteritidis | 2-12-3-3-NA | YES | YES | ERS4406535 | ST11 | YES | 0 |
|  | KST1442 | Stool | 2015 | 1 | *Salmonella* Enteritidis | 2-12-3-3-NA |  | YES | ERS4406591 | ST11 |  |  |
| 30 | 12046/3 | Blood | 2015 | NA | *Salmonella* Typhimurium | 2-7-9-9-0210 | YES | YES | ERS4406536 | ST313 | YES | 1 |
|  | KST1464 | Stool | 2015 | 3 | *Salmonella* Typhimurium | 2-7-9-9-0210 |  | YES | ERS4406594 | ST313 |  |  |
| 31 | 12174/3 | Blood | 2015 | NA | *Salmonella* Typhimurium | 2-8-12-8-0210 | NO | YES | ERS4406537 | ST313 | NO | 45 |
|  | KST1482 | Stool | 2015 | 1 | *Salmonella* Typhimurium | 3-NA-10-7-0210 |  | YES | ERS4406595 | ST313 |  |  |
| 32 | 12310/3 | Blood | 2015 | NA | *Salmonella* Enteritidis | 2-18-3-3-NA | YES | NO | NA | ST11 | NA | NA |
|  | KST1516 | Stool | 2015 | 1 | *Salmonella* Enteritidis | 2-18-3-3-NA |  | NO | NA | ST11 |  |  |
| 33 | 12306/3 | Blood | 2015 | NA | *Salmonella* Typhimurium | 2-6-9-9-0210 | YES | YES | ERS4406538 | ST313 | YES | 1 |
|  | KST1518 | Stool | 2015 | 1 | *Salmonella* Typhimurium | 2-6-9-9-0210 |  | YES | ERS4406597 | ST313 |  |  |
| 34 | 12434/3 | Blood | 2015 | NA | *Salmonella* Typhimurium | 3-NA-10-7-0210 | YES | YES | ERS4406539 | ST313 | YES | 2 |
|  | KST1535 | Stool | 2015 | 2 | *Salmonella* Typhimurium | 3-NA-10-7-0210 |  | YES | ERS4406596 | ST313 |  |  |
| 35 | 12727/3 | Blood | 2015 | NA | *Salmonella* Typhimurium | 2-6-9-9-0210 | YES | YES | ERS4406540 | ST313 | YES | 2 |
|  | KST1588 | Stool | 2015 | 4 | *Salmonella* Typhimurium | 2-6-9-9-0210 |  | YES | ERS4406598 | ST313 |  |  |
| 36 | 13861/3 | Blood | 2015 | NA | *Salmonella* Typhimurium | 2-6-9-9-0210 | YES | NO | NA | ST313 | NA | NA |
|  | KST1873 | Stool | 2015 | 0 | *Salmonella* Typhimurium | 2-6-9-9-0210 |  | NO | NA | ST313 |  |  |
| 37 | 13894/3 | Blood | 2015 | NA | *Salmonella* Typhimurium | 2-5-10-7-0210 | YES | YES | ERS4406541 | ST313 | YES | 1 |
|  | KST1893 | Stool | 2015 | 2 | *Salmonella* Typhimurium | 2-5-10-7-0210 |  | YES | ERS4406599 | ST313 |  |  |
| 38 | 13935/3 | Blood | 2015 | NA | *Salmonella* Enteritidis | 2-13-3-3-NA | YES | YES | ERS4406542 | ST11 | YES | 3 |
|  | KST1911 | Stool | 2015 | 2 | *Salmonella* Enteritidis | 2-13-3-3-NA |  | YES | ERS4406601 | ST11 |  |  |
| 39 | 14028/3 | Blood | 2015 | NA | *Salmonella* Enteritidis | 2-15-3-3-NA | YES | YES | ERS4406543 | ST11 | YES | 1 |
|  | KST1927 | Stool | 2015 | 1 | *Salmonella* Enteritidis | 2-15-3-3-NA |  | YES | ERS4406600 | ST11 |  |  |
| 40 | 14867/3 | Blood | 2015 | NA | *Salmonella* Enteritidis | 2-13-3-3-NA | YES | YES | ERS4406544 | ST11 | YES | 1 |
|  | KST2150 | Stool | 2015 | 2 | *Salmonella* Enteritidis | 2-13-3-3-NA |  | YES | ERS4406602 | ST11 |  |  |
| 41 | 15139/3 | Blood | 2015 | NA | *Salmonella* Typhimurium | 2-9-12-7-0210 | YES | YES | ERS4406545 | ST313 | YES | 3 |
|  | KST2171 | Stool | 2015 | 3 | *Salmonella* Typhimurium | 2-9-12-7-0210 |  | YES | ERS4406603 | ST313 |  |  |
| 42 | 15401/3 | Blood | 2015 | NA | *Salmonella* Enteritidis | 2-18-3-3-NA | YES | YES | ERS4406546 | ST11 | YES | 0 |
|  | KST2183 | Stool | 2015 | 1 | *Salmonella* Enteritidis | 2-18-3-3-NA |  | YES | ERS4406605 | ST11 |  |  |
| 43 | 15560/3 | Blood | 2015 | NA | *Salmonella* Typhimurium | 2-6-9-9-0210 | YES | YES | ERS4406547 | ST313 | NO | 28 |
|  | KST2195 | Stool | 2015 | 6 | *Salmonella* Typhimurium | 2-6-9-9-0210 |  | YES | ERS4406604 | ST313 |  |  |
| 44 | 15826/3 | Blood | 2016 | NA | *Salmonella* Typhimurium | 2-6-9-9-0210 | YES | YES | ERS4406548 | ST313 | NO | 3 |
|  | KST2249 | Stool | 2016 | 1 | *Salmonella* Typhimurium | 2-6-9-9-0210 |  | YES | ERS4406606 | ST313 |  |  |
| 45 | 16037/3 | Blood | 2016 | NA | *Salmonella* Typhimurium | 2-6-9-9-0210 | YES | YES | ERS4406549 | ST313 | YES | 0 |
|  | KST2276 | Stool | 2016 | 1 | *Salmonella* Typhimurium | 2-6-9-9-0210 |  | YES | ERS4406607 | ST313 |  |  |
| 46 | 16165/3 | Blood | 2016 | NA | *Salmonella* Typhimurium | 2-5-14-8-0210 | YES | YES | ERS4406550 | ST313 | YES | 2 |
|  | KST2340 | Stool | 2016 | 2 | *Salmonella* Typhimurium | 2-5-14-8-0210 |  | YES | ERS4406608 | ST313 |  |  |
| 47 | 16371/3 | Blood | 2016 | NA | *Salmonella* Typhimurium | 2-6-9-9-0210 | YES | YES | ERS4406551 | ST313 | YES | 3 |
|  | KST2459 | Stool | 2016 | 1 | *Salmonella* Typhimurium | 2-6-9-9-0210 |  | YES | ERS4406609 | ST313 |  |  |
| 48 | 16386/3 | Blood | 2016 | NA | *Salmonella* Typhimurium | 2-6-9-9-0210 | YES | YES | ERS4406552 | ST313 | YES | 0 |
|  | KST2460 | Stool | 2016 | 1 | *Salmonella* Typhimurium | 2-6-9-9-0210 |  | YES | ERS4406610 | ST313 |  |  |
| 49 | 16463/3 | Blood | 2016 | NA | *Salmonella* Typhimurium | 2-6-9-9-0210 | YES | YES | ERS4406553 | ST313 | YES | 5 |
|  | KST2485 | Stool | 2016 | 1 | *Salmonella* Typhimurium | 2-6-9-9-0210 |  | YES | ERS4406611 | ST313 |  |  |
| 50 | 16690/3 | Blood | 2016 | NA | *Salmonella* Typhimurium | 2-6-9-9-0210 | YES | NO | NA | ST313 | NA | NA |
|  | KST2543 | Stool | 2016 | 8 | *Salmonella* Typhimurium | 2-6-9-9-0210 |  | NO | NA | ST313 |  |  |
| 51 | 16812/3 | Blood | 2016 | NA | *Salmonella* Typhimurium | 2-6-9-9-0210 | YES | YES | ERS4406555 | ST313 | YES | 1 |
|  | KST2563 | Stool | 2016 | 1 | *Salmonella* Typhimurium | 2-6-9-9-0210 |  | YES | ERS4406612 | ST313 |  |  |
| 52 | 16884/3 | Blood | 2016 | NA | *Salmonella* Typhimurium | 2-6-9-9-0210 | YES | YES | ERS4406554 | ST313 | YES | 0 |
|  | KST2587 | Stool | 2016 | 1 | *Salmonella* Typhimurium | 2-6-9-9-0210 |  | YES | ERS4406613 | ST313 |  |  |
| 53 | 17120/3 | Blood | 2016 | NA | *Salmonella* Enteritidis | 2-17-3-3-NA | YES | YES | ERS4406556 | ST11 | YES | 0 |
|  | KST2628 | Stool | 2016 | 1 | *Salmonella* Enteritidis | 2-17-3-3-NA |  | YES | ERS4406614 | ST11 |  |  |
| 54 | 17064/3 | Blood | 2016 | NA | *Salmonella* Typhimurium | 2-6-9-9-0210 | YES | YES | ERS4406558 | ST313 | YES | 0 |
|  | KST2635 | Stool | 2016 | 5 | *Salmonella* Typhimurium | 2-6-9-9-0210 |  | YES | ERS4406615 | ST313 |  |  |
| 55 | 17918/3 | Blood | 2016 | NA | *Salmonella* Enteritidis | 2-17-3-3-NA | YES | NO | NA | ND | NA | NA |
|  | KST2977 | Stool | 2016 | 2 | *Salmonella* Enteritidis | 2-17-3-3-NA |  | NO | NA | ND |  |  |
| 56 | 18074/3 | Blood | 2016 | NA | *Salmonella* Enteritidis | 2-15-3-3-NA | YES | NO | NA | ND | NA | NA |
|  | KST3018 | Stool | 2016 | 1 | *Salmonella* Enteritidis | 2-15-3-3-NA |  | NO | NA | ND |  |  |
| 57 | 18198/3 | Blood | 2016 | NA | *Salmonella* Typhimurium | 2-6-9-9-0210 | YES | NO | NA | ND | NA | NA |
|  | KST3104 | Stool | 2016 | 4 | *Salmonella* Typhimurium | 2-6-9-9-0210 |  | NO | NA | ND |  |  |
| 58 | 18219/3 | Blood | 2016 | NA | *Salmonella* Typhimurium | 2-6-9-9-0210 | YES | NO | NA | ND | NA | NA |
|  | KST3111 | Stool | 2016 | 4 | *Salmonella* Typhimurium | 2-6-9-9-0210 |  | NO | NA | ND |  |  |
| 59 | 18453/3 | Blood | 2016 | NA | *Salmonella* Typhimurium | 2-NA-9-7-0210 | YES | NO | NA | ND | NA | NA |
|  | KST3154 | Stool | 2016 | 2 | *Salmonella* Typhimurium | 2-NA-9-7-0210 |  | NO | NA | ND |  |  |
| 60 | 18630/3 | Blood | 2016 | NA | *Salmonella* Typhimurium | 2-5-15-8-0210 | YES | NO | NA | ND | NA | NA |
|  | KST3187 | Stool | 2016 | 1 | *Salmonella* Typhimurium | 2-5-15-8-0210 |  | NO | NA | ND |  |  |
| 61 | 18711/3 | Blood | 2016 | NA | *Salmonella* Typhimurium | 2-8-12-9-0210 | YES | NO | NA | ND | NA | NA |
|  | KST3199 | Stool | 2016 | 1 | *Salmonella* Typhimurium | 2-8-12-9-0210 |  | NO | NA | ND |  |  |
| 62 | 18809/3 | Blood | 2016 | NA | *Salmonella* Typhimurium | 2-7-15-6-0210 | YES | NO | NA | ND | NA | NA |
|  | KST3219 | Stool | 2016 | 1 | *Salmonella* Typhimurium | 2-7-15-6-0210 |  | NO | NA | ND |  |  |
| 63 | 19523/3 | Blood | 2016 | NA | *Salmonella* Typhimurium | 2-5-15-8-0210 | NO | NO | NA | ND | NA | NA |
|  | KST3269 | Stool | 2016 | 4 | *Salmonella* Typhimurium | 2-8-10-8-0210 |  | NO | NA | ND |  |  |
| 64 | 19015/3 | Blood | 2016 | NA | *Salmonella* Typhimurium | 2-5-13-8-0210 | YES | NO | NA | ND | NA | NA |
|  | KST3412 | Stool | 2016 | 16 | *Salmonella* Typhimurium | 2-5-13-8-0210 |  | NO | NA | ND |  |  |
| 65 | 19351/3 | Blood | 2016 | NA | *Salmonella* Typhimurium | 2-6-9-9-0210 | YES | NO | NA | ND | NA | NA |
|  | KST3440 | Stool | 2016 | 4 | *Salmonella* Typhimurium | 2-6-9-9-0210 |  | NO | NA | ND |  |  |
| 66 | 19499/3 | Blood | 2016 | NA | *Salmonella* Typhimurium | 2-6-9-9-0210 | YES | NO | NA | ND | NA | NA |
|  | KST3461 | Stool | 2016 | 3 | *Salmonella* Typhimurium | 2-6-9-9-0210 |  | NO | NA | ND |  |  |
| 67 | 19681/3 | Blood | 2016 | NA | *Salmonella* Enteritidis | 2-15-3-3-NA | YES | NO | NA | ND | NA | NA |
|  | KST3499 | Stool | 2016 | 2 | *Salmonella* Enteritidis | 2-15-3-3-NA |  | NO | NA | ND |  |  |
| 68 | 19818/3 | Blood | 2016 | NA | *Salmonella* Typhimurium | 2-NA-9-7-0210 | YES | NO | NA | ND | NA | NA |
|  | KST3520 | Stool | 2016 | 1 | *Salmonella* Typhimurium | 2-NA-9-7-0210 |  | NO | NA | ND |  |  |
| 69 | 19804/3 | Blood | 2016 | NA | *Salmonella* Enteritidis | 2-15-3-3-NA | YES | NO | NA | ND | NA | NA |
|  | KST3524 | Stool | 2016 | 3 | *Salmonella* Enteritidis | 2-15-3-3-NA |  | NO | NA | ND |  |  |
| 70 | 19828/3 | Blood | 2016 | NA | *Salmonella* Typhimurium | 2-9-12-7-0210 | YES | NO | NA | ND | NA | NA |
|  | KST3533 | Stool | 2016 | 1 | *Salmonella* Typhimurium | 2-9-12-7-0210 |  | NO | NA | ND |  |  |
| 71 | 20083/3 | Blood | 2017 | NA | *Salmonella* Typhimurium | 2-5-15-8-0210 | YES | NO | NA | ND | NA | NA |
|  | KST3591 | Stool | 2017 | 2 | *Salmonella* Typhimurium | 2-5-15-8-0210 |  | NO | NA | ND |  |  |
| 72 | 20175/3 | Blood | 2017 | NA | *Salmonella* Typhimurium | 2-5-15-8-0210 | NO | NO | NA | ND | NA | NA |
|  | KST3610 | Stool | 2017 | 6 | *Salmonella* Typhimurium | 2-4-12-7-0210 |  | NO | NA | ND |  |  |
| 73 | 20195/3 | Blood | 2017 | NA | *Salmonella* Typhimurium | 2-NA-9-7-0210 | YES | NO | NA | ND | NA | NA |
|  | KST3615 | Stool | 2017 | 1 | *Salmonella* Typhimurium | 2-NA-9-7-0210 |  | NO | NA | ND |  |  |
| 74 | 20328/3 | Blood | 2017 | NA | *Salmonella* Typhimurium | 2-5-16-8-0210 | NO | NO | NA | ND | NA | NA |
|  | KST3635 | Stool | 2017 | 2 | *Salmonella* Typhimurium | 2-8-11-NA-0210 |  | NO | NA | ND |  |  |
| 75 | 20355/3 | Blood | 2017 | NA | *Salmonella* Typhimurium | 2-8-12-8-0210 | YES | NO | NA | ND | NA | NA |
|  | KST3638 | Stool | 2017 | 2 | *Salmonella* Typhimurium | 2-8-12-8-0210 |  | NO | NA | ND |  |  |
| 76 | 20324/3 | Blood | 2017 | NA | *Salmonella* Typhimurium | 2-NA-9-7-0210 | YES | NO | NA | ND | NA | NA |
|  | KST3646 | Stool | 2017 | 5 | *Salmonella* Typhimurium | 2-NA-9-7-0210 |  | NO | NA | ND |  |  |
| 77 | 20461/3 | Blood | 2017 | NA | *Salmonella* Typhimurium | 2-9-11-7-0210 | YES | NO | NA | ND | NA | NA |
|  | KST3670 | Stool | 2017 | 2 | *Salmonella* Typhimurium | 2-9-11-7-0210 |  | NO | NA | ND |  |  |
| 78 | 20538/3 | Blood | 2017 | NA | *Salmonella* Typhimurium | 2-8-12-8-0210 | YES | NO | NA | ND | NA | NA |
|  | KST3681 | Stool | 2017 | 2 | *Salmonella* Typhimurium | 2-8-12-8-0210 |  | NO | NA | ND |  |  |
| 79 | 20523/3 | Blood | 2017 | NA | *Salmonella* Enteritidis | 2-13-3-3-NA | YES | NO | NA | ND | NA | NA |
|  | KST3682 | Stool | 2017 | 3 | *Salmonella* Enteritidis | 2-13-3-3-NA |  | NO | NA | ND |  |  |
| 80 | 20908/3 | Blood | 2017 | NA | *Salmonella* Typhimurium | 2-NA-9-7-0210 | YES | NO | NA | ND | NA | NA |
|  | KST3748 | Stool | 2017 | 2 | *Salmonella* Typhimurium | 2-NA-9-7-0210 |  | NO | NA | ND |  |  |
| 81 | 21013/3 | Blood | 2017 | NA | *Salmonella* Typhimurium | 2-5-15-8-0210 | YES | NO | NA | ND | NA | NA |
|  | KST3762 | Stool | 2017 | 1 | *Salmonella* Typhimurium | 2-5-15-8-0210 |  | NO | NA | ND |  |  |
| 82 | 21024/3 | Blood | 2017 | NA | *Salmonella* Typhimurium | 2-5-10-7-0210 | YES | NO | NA | ND | NA | NA |
|  | KST3776 | Stool | 2017 | 5 | *Salmonella* Typhimurium | 2-5-10-7-0210 |  | NO | NA | ND |  |  |
| 83 | 21228/3 | Blood | 2017 | NA | *Salmonella* Typhimurium | 2-5-15-8-0210 | YES | NO | NA | ND | NA | NA |
|  | KST3809 | Stool | 2017 | 2 | *Salmonella* Typhimurium | 2-5-15-8-0210 |  | NO | NA | ND |  |  |
| 84 | 21188/3 | Blood | 2017 | NA | *Salmonella* Typhimurium | 2-5-15-8-0210 | YES | NO | NA | ND | NA | NA |
|  | KST3819 | Stool | 2017 | 8 | *Salmonella* Typhimurium | 2-5-15-8-0210 |  | NO | NA | ND |  |  |
| 85 | 21332/3 | Blood | 2017 | NA | *Salmonella* Typhimurium | 2-5-13-8-0210 | YES | NO | NA | ND | NA | NA |
|  | KST3833 | Stool | 2017 | 2 | *Salmonella* Typhimurium | 2-5-13-8-0210 |  | NO | NA | ND |  |  |
| 86 | 21308/3 | Blood | 2017 | NA | *Salmonella* Typhimurium | 2-6-9-9-0210 | YES | NO | NA | ND | NA | NA |
|  | KST3838 | Stool | 2017 | 4 | *Salmonella* Typhimurium | 2-6-9-9-0210 |  | NO | NA | ND |  |  |
| 87 | 21494/3 | Blood | 2017 | NA | *Salmonella* Typhimurium | 2-5-16-8-0210 | YES | NO | NA | ND | NA | NA |
|  | KST3867 | Stool | 2017 | 1 | *Salmonella* Typhimurium | 2-5-16-8-0210 |  | NO | NA | ND |  |  |

Abbreviations: MLVA = multiple-locus variable-number of tandem repeats analysis, MLST = multi-locus sequence type, ND = no data, NA = not applicable, SNP = single nucleotide polymorphism, WGS = whole genome sequencing.
