# Supplementary material for: High genetic similarity between non-typhoidal Salmonella isolated from paired blood and stool samples of children in the Democratic Republic of the Congo
Source: PLoS Negl Trop Dis. 2020 Jul 2;14(7):e0008377. doi: 10.1371/journal.pntd.0008377 (PMC7331982; doi:10.1371/journal.pntd.0008377)
Supplement: S3 Table — Abbreviations: MLVA = multiple-locus variable-number of tandem repeats analysis, NA = not applicable, NTS = non-typhoidal Salmonella. (DOCX) [file pntd.0008377.s004.docx]

**S3 Table. Overview of MLVA types of NTS stool isolates recovered from the control group.**

| **Typhimurium MLVA types from stool** | **N control group** |
| --- | --- |
| 2-4-12-7-0210 | 1 |
| 2-4-15-8-0210 | 1 |
| 2-5-10-7-0210 | 1 |
| 2-5-12-10-0210 | 1 |
| 2-5-14-8-0210 | 1 |
| 2-5-15-8-0210 | 1 |
| 2-6-9-9-0210 | 6 |
| 2-7-14-6-0210 | 1 |
| 2-7-15-6-0210 | 1 |
| 2-8-5-NA-0210 | 1 |
| 2-8-9-8-0210 | 2 |
| 2-8-10-8-0210 | 2 |
| 2-9-11-7-0210 | 1 |
| 2-9-12-NA-0210 | 1 |
| 2-17-14-8-0210 | 1 |
| 2-NA-12-7-0210 | 1 |
| 2-NA-15-8-0210 | 1 |
| 3-NA-12-7-0210 | 1 |
| Total | 25 |
| **Enteritidis MLVA types from stool** | **N control group** |
| 2-10-7-3-2 | 1 |
| 2-13-3-3-NA | 3 |
| 2-15-3-3-NA | 2 |
| 2-17-3-3-NA | 1 |
| 2-18-3-3-NA | 2 |
| Total | 9 |

Abbreviations: MLVA = multiple-locus variable-number of tandem repeats analysis, NA = not applicable, NTS = non-typhoidal *Salmonella.*
