# Supplementary figures and images for: High genetic similarity between non-typhoidal Salmonella isolated from paired blood and stool samples of children in the Democratic Republic of the Congo
Source: PLoS Negl Trop Dis. 2020 Jul 2;14(7):e0008377. doi: 10.1371/journal.pntd.0008377 (PMC7331982; doi:10.1371/journal.pntd.0008377)

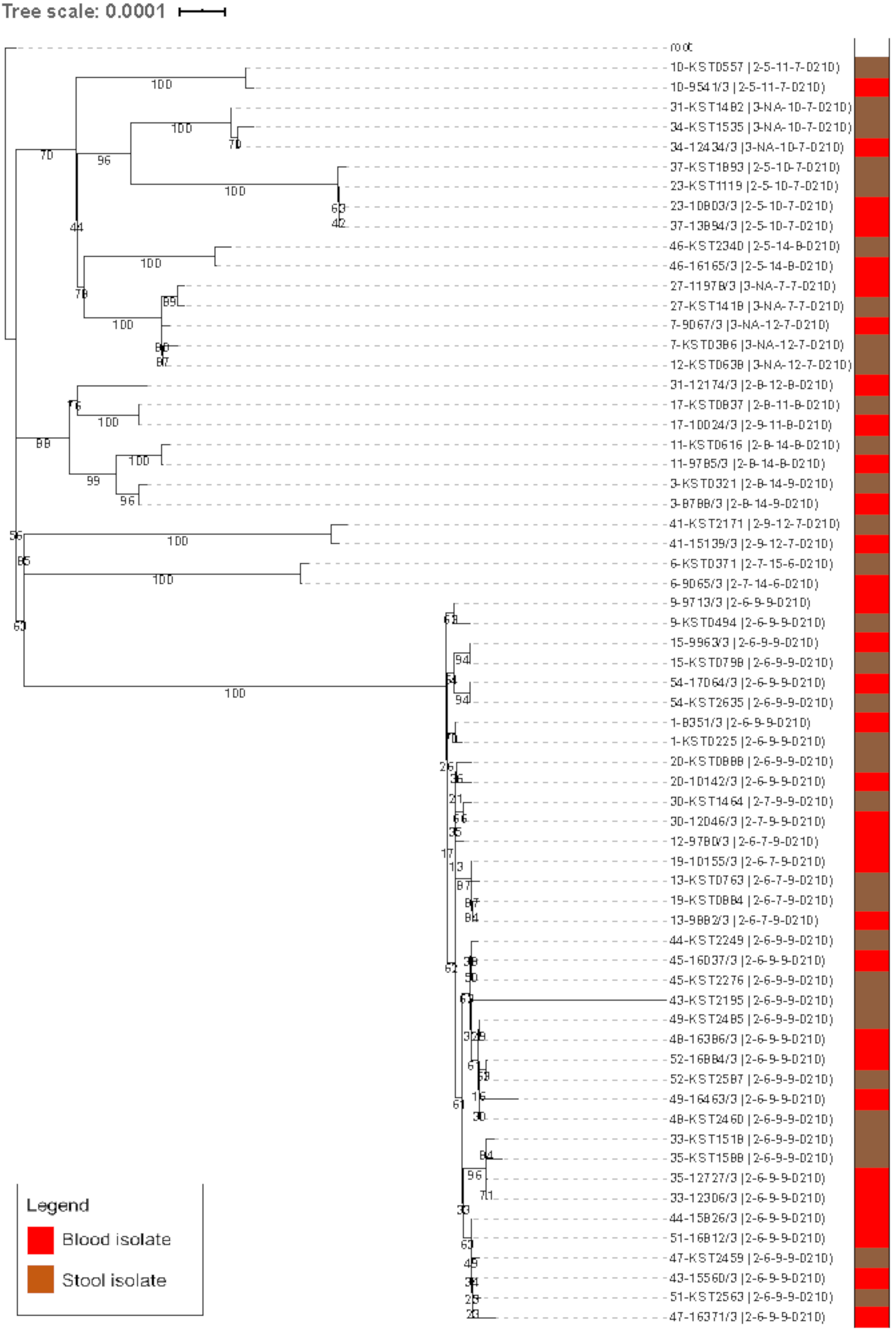

Supplement: S1 Fig — Maximum likelihood phylogenetic tree of Salmonella Typhimurium paired isolates, mapped on reference strain Salmonella Typhimurium D23580 (Accession Number = NC_016854) and rooted on Salmonella Typhi 10040_15 (Accession Number = ERS1574281). The bootstrap values of the branch nodes are indicated on the branches. The branch lengths represent the nucleotide substitution rate in the core SNP alignment of 58148 SNPs. The isolate names have a prefix representing the pair number followed by the study number of each isolate (S2 Table) and a suffix between brackets indicating the MVLA type. The bar on the right indicates the specimen of isolation (blood isolate (red) versus stool isolate (brown)). (TIF) [file pntd.0008377.s005.tif]
